# Masticatory jaw movement of *Exaeretodon argentinus* (Therapsida: Cynodontia) inferred from its dental microwear

**DOI:** 10.1371/journal.pone.0188023

**Published:** 2017-11-29

**Authors:** Tai Kubo, Eisuke Yamada, Mugino O. Kubo

**Affiliations:** 1 The University Museum, The University of Tokyo, Hongo 7-3-1, Bunkyo-ku, Tokyo, Japan; 2 Department of Evolutionary Studies of Biosystems, School of Advanced Sciences, SOKENDAI (The Graduate University for Advanced Studies), Shonan Village, Hayama, Kanagawa, Japan; 3 Department of Natural Environmental Studies, Graduate School of Frontier Sciences, The University of Tokyo, Kashiwanoha 5-1-5, Kashiwa, Chiba, Japan; Monash University, AUSTRALIA

## Abstract

Dental microwear of four postcanine teeth of *Exaeretodon argentinus* was analyzed using both two dimensional (2D) and three dimensional (3D) methods to infer their masticatory jaw movements. Results of both methods were congruent, showing that linear microwear features (scratches) were well aligned and mostly directed to the antero-posterior direction in all four teeth examined. These findings support the palinal masticatory jaw movement, which was inferred in previous studies based on the observation of gross morphology of wear facets. In contrast, the lack of detection of lateral scratches confirmed the absence of the lateral jaw movement that was also proposed by a previous study. Considering previous microwear studies on cynodonts, palinal jaw movements observed in *Exaeretodon* evolved within cynognathian cynodonts from the fully orthal jaw movement of its basal member. Although there are currently only three studies of dental microwear of non-mammalian cynodonts including the present study, microwear analysis is a useful tool for the reconstruction of masticatory jaw movement and its future application to various cynodonts will shed light on the evolutionary process of jaw movement towards the mammalian condition in more detail.

## Introduction

Evolution of postcanine occlusal pattern and masticatory jaw movement toward the mammalian condition among cynodonts has been studied intensively as one of the historical topics in vertebrate paleontology [1–3]. The mammalian condition, which enabled precise occlusion of upper and lower postcanines and jaw movement only on one side, increased their bite force and consequently contributed to their success [1]. Further, masticatory jaw movements of cynodonts had been considered to have some phylogenetic significance; for example, palinal (i.e., backward) movement of the mandible of traversodontids and tritylodontids was considered evidence of their ancestor-descendant relationship [2]. However, due to the recent advances in understanding of the phylogeny of cynodonts [4], it is now considered that various jaw movement patterns of cynodonts were evolved as an adaptation for various diets. Palinal jaw movements were perhaps adaptations for an herbivorous diet, which evolved independently among traversodontids and tritylodontids, and also within mammaliaforms among haramiyidans and multituberculates [3].

Occlusal patterns and jaw movements of non-mammalian cynodonts and early mammals have been largely inferred from the gross morphology of teeth by examining how cusps, ridges and basins of antagonistic teeth occluded with each other during mastication [2,3,5,6]. Based on these simple observation, jaw movements of *Exaeretodon*, a derived traversodontid that was found mostly from Late Triassic strata of Argentina and Brazil [7], was studied by Crompton [2] and Goñi and Goin [6]. Both studies assumed bilateral movement of mandible, in other words, there is no mobility at the mandibular symphysis and occlusion occurred simultaneously in postcanine tooth rows of both mandibular rami. Both studies also assumed after the occlusion of upper and lower postcanines by orthal movement, palinal movement occurred as a masticatory jaw movement, which pulled the mandible backward against the upper jaw [2,6]. Goñi and Goin [6], but not Crompton[2], assumed after these orthal and palinal jaw movements, transverse movement occurred, where lower postcanines move medially against upper postcanines.

Microwear analysis examines microscopic scars (e.g. pits and scratches) on tooth enamel surfaces that were produced during mastication. Historically, the study of dental microwear began for the purpose of reconstructing jaw movement, because the directions of scratches are likely to reflect the relative direction of jaw movement [8,9]. This inference was confirmed through experimental study on extant mammals [10]. It is now widely accepted that the direction of scratches at occlusal surfaces reflects the relative movement of antagonistic teeth and direction of jaw movement can be reconstructed by integrating it with other information such as tooth morphology and tooth position in relation to the skull. Along with diet, jaw movement was often considered as the main information that can be revealed by dental microwear analysis [11]. This method was widely used in reconstructing the jaw movements of Mesozoic tetrapods [12–16], however, tooth microwear of non-mammalian cynodonts has been examined only in two studies so far [15,17]. Grine [17] examined microwear of a basal cynognathian cynodont, *Diademodon*, and found pitted tooth surface texture that indicate direct pounding between upper and lower teeth due to the wholly orthal jaw movement. Goswami [15] showed that the microwear of the traversodontid, *Dadadon isaloi*, and two unnamed traversodontid specimens were dominated by scratches, which were oriented in a bimodal manner. Scratches of both upper and lower teeth predominantly run in a dorso-posterior orientation. Upper teeth display a second dominant orientation of scratches in an antero-posterior orientation, whereas the second dominant orientation of lower teeth is in the dorso-ventral orientation. These scratches were interpreted as the result of orthal and palinal jaw movements.

The aim of this study is to analyze dental microwear of *Exaeretodon argentines* to reconstruct its jaw movement and compare the results with previous reconstructions based on gross morphology of wear facets [2,6]. The direction of the masticatory jaw movement was reconstructed by measuring the orientation of scratches from two dimensional images of tooth surfaces and also by calculating the direction of surface texture anisotropy obtained from 3D surface data of dental microwear.

## Materials and methods

**Fig 1:**
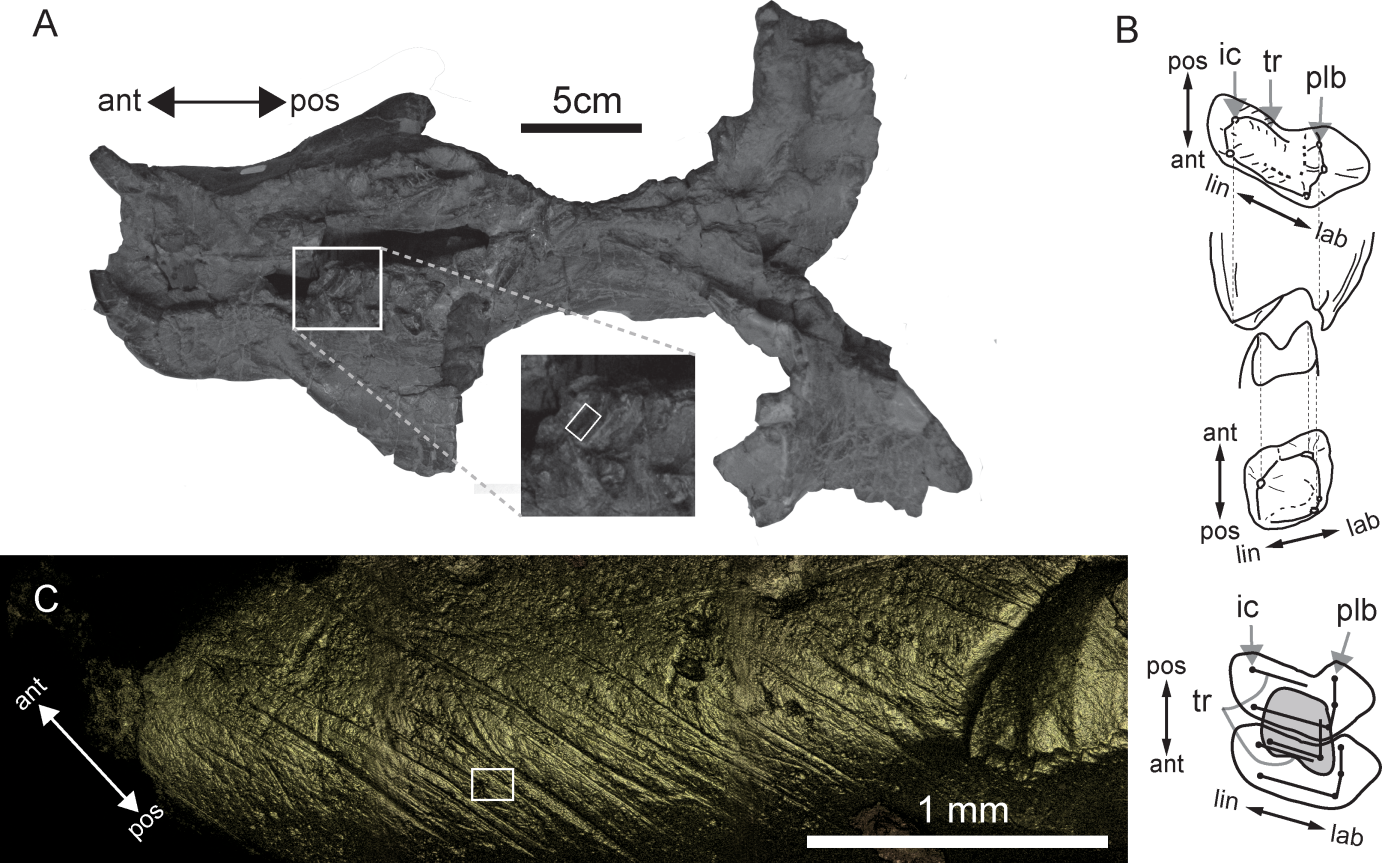
Skull and postcanine teeth of *Exaeretodon argentinus* PVSJ 1091 and its dental microwear. (A) Palatal view of the skull of *Exaeretodon argentines* PVSJ 1091. White rectangle in the close-up photo on the labial side of the transverse ridge shows where the tooth mold was taken for the dental microwear analysis. (B) From top to bottom, occlusal view of a right upper postcanine tooth, posterior view of a right upper postcanine tooth, posterior view of a right lower postcanine tooth, and occlusal view of a right lower postcanine tooth. Corresponding cusps are connected by dotted lines. At the bottom, a schematic diagram showing positions of two upper postcanine teeth and one lower postcanine tooth (gray) in occlusion. From this position, the lower postcanine was pulled posteriorly so that its mesial ridge was dragged against the upper postcanine during the masticatory movement, against the transverse ridge of the anterior upper postcanine to the mesial ridge of the adjacent posterior upper postcanine. Black straight lines represent ridges and black circles represent cusps. Modified from [2,27]. (C) Surface image of the transverse ridge of the second postcanine of PVSJ 1091. This image was taken from the mold using a confocal laser microscope (VK-9700) with a 10× objective lens. The image was mirrored in the horizontal direction to match its direction with the real tooth surface. A white rectangle shows the area where 3D coordinates were taken with a 100× objective lens to obtain ISO 25178 parameters (Fig 3). ant, anterior; ic, internal cusp; lab, labial; lin, lingual; plb, posterior labial cusp; pos, posterior; tr, transverse ridge.

**Fig 2:**
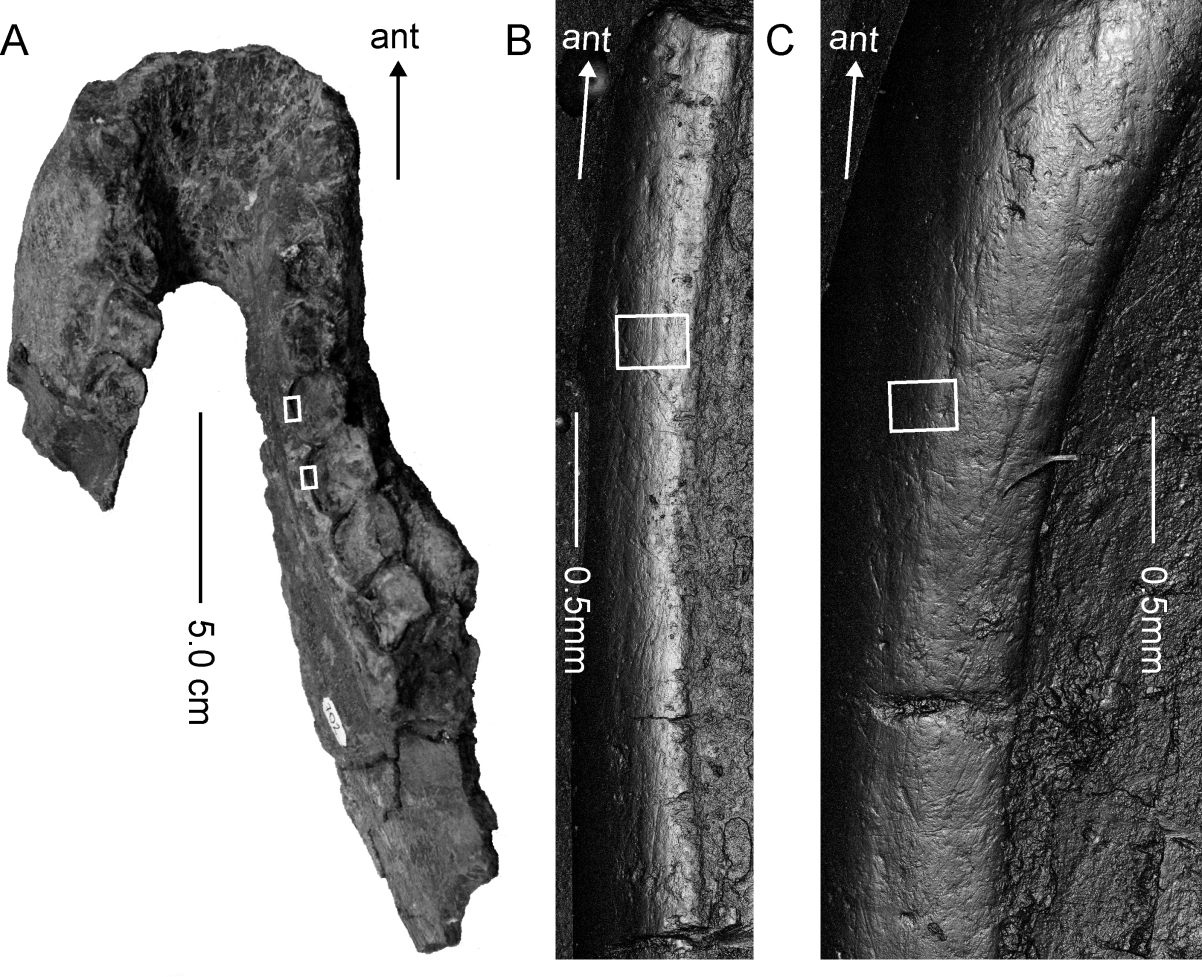
Mandible and postcanine teeth of *Exaeretodon argentinus* PVSJ 702 and its dental microwear. (A) Occlusal view of the mandible of *Exaeretodon argentines* PVSJ 702. The white rectangle represents the area from where tooth molds were taken for the dental microwear analysis. (B) and (C) Surface images of the lingual sides of the third and fourth postcanines of PVSJ702, respectively. These images were taken from molds using a confocal laser microscope (VK-9700) with a 50× objective lens. Images were mirrored in the horizontal direction to match its direction with the real tooth surfaces. White rectangles show areas where 3D coordinates were taken with a 100× objective lens to obtain ISO 25178 parameters.

Molds were taken from 13 teeth of six *Exaeretodon argentines* specimens stored in the Instituto y Museo de Ciencias Naturales of the Universidad Nacional de San Juan (PVSJ). Specimen numbers are PVSJ 74, PVSJ 109, PVSJ 623, PVSJ 702, PVSJ 707, and PVSJ 1091. These *Exaeretodon* specimens were collected from the Upper Triassic Ischigualasto formation (Carnian-Norian) in San Juan Province, northwestern Argentina [18]. The tooth surfaces of these specimens were examined using a stereomicroscope to locate well preserved shiny enamel regions. These surfaces were cleaned using acetone and cotton swabs and molded using Affinis Regular Body polyvinylsiloxane (Coltène Whaledent Inc. Altstätten, Switzerland) that has a medium viscosity and medium consistency (ISO4823, Type2). The obtained dental impressions were scanned using a confocal laser microscope (VK-9700, Keyence Co, Japan) using a violet laser (wavelength 408nm) with 10× and 50× objective lens (numerical apertures 0.30 and 0.55 respectively) to obtain surface images. On each of the XY coordinate points, light intensity of reflected laser, color information and height data (Z position) were obtained at the focus point. Using these data the VK-9700 device generates real color ultra-depth images. By examining images of mold surfaces, preservation of microwear was confirmed for four specimens. Specimen number and anatomical positions of these four specimens are: the posterior edge (hereafter referred as transverse ridge following [2]; see Fig 1B for its position) of the second postcanine tooth of right upper jaw of PVSJ 1091 (Fig 1); the posterior lingual cusp (hereafter referred as internal cusp following [2]; see Fig 1B for its position) of the second postcanine tooth of left upper jaw of PVSJ 707; and lingual side of occlusal surfaces of third and fourth postcanine teeth of right mandible of PVSJ 702 (Fig 2). Among these four tooth surfaces, the internal cusp of the second postcanine of PVSJ 707 is not occluding surface, because upper postcanines of *Exaeretodon* were medio-laterally longer than antagonistic lower postcanines and occluded only at the labio-lingual middle region (Fig 1B). These specimens must have been affected by taphonomic processes to some extent as cracks were observed on tooth surfaces (Figs 1 and 2). Taphonomic processes, however, tend to obliterate rather than modify dental microwear [19], and as shown in King et al. [19] orientation and length of scratches, that were used in our analyses, were unaffected by taphonomy, if not obliterated.

Total skull lengths of PVSJ 1091 and 707 were approximately 30 cm and 29 cm, respectively, whereas PVSJ 702 was too fragmented to infer size of the mandible (Fig 2). Among 12 specimens of *Exaeretodon* including PVSJ 707, for which skull length was measured by Liu [20], PVSJ 707 is the fifth smallest (eighth largest), and PVSJ 1091 is between fifth and sixth smallest specimens. The growth pattern of *Exaeretodon* and sexual material are not known, but from the skull sizes it appears that PVSJ 1091 and 707 are probably adults and not juveniles.

Images for 2D dental microwear analyses were obtained using a 10× objective lens for PVSJ 1091 and a 50× objective lens for other specimens with a scanning pitch of 1 μm in the Z-axis. To analyze dental microwear, scanned images of tooth surfaces were combined to compose images covering the area where microwear is preserved. This combined image was mirrored because the mold is a mirror image of the real tooth surface. Analyzed images covered an area of 3837×1045 μm for the second postcanine of PVSJ 1091 (Fig 1C), 737×3571 μm for the third postcanine of PVSJ 702 (Fig 2B), 1487×3740 μm for the fourth postcanine of PVSJ 702 (Fig 2C), and 760×1452 μm for the second postcanine of PVSJ 707. Angle and length of linear microwear (scratches) were measured using the image processing software Image J [21]. For slightly curved scratches, the two end points were connected to measure the angle and length. Some scratches were truncated by postmortem damage and such scratches were not measured. Mean orientation (mean vector), 95% bootstrap confidence intervals, angular dispersion, mean scratch lengths, and 95% bootstrap confidence intervals were calculated for scratches in each specimen. Angular dispersion represents a degree of parallelism, so if scratches are aligned, the angular dispersion approaches a value of one, and if scratches are oriented more randomly then it approaches zero [16]. Bootstrap confidence intervals were calculated using the packages “circular” and “boot” of software “R” [22]. The software “Rose” version 2.1 [23] was used to draw Rose diagrams.

**Fig 3:**
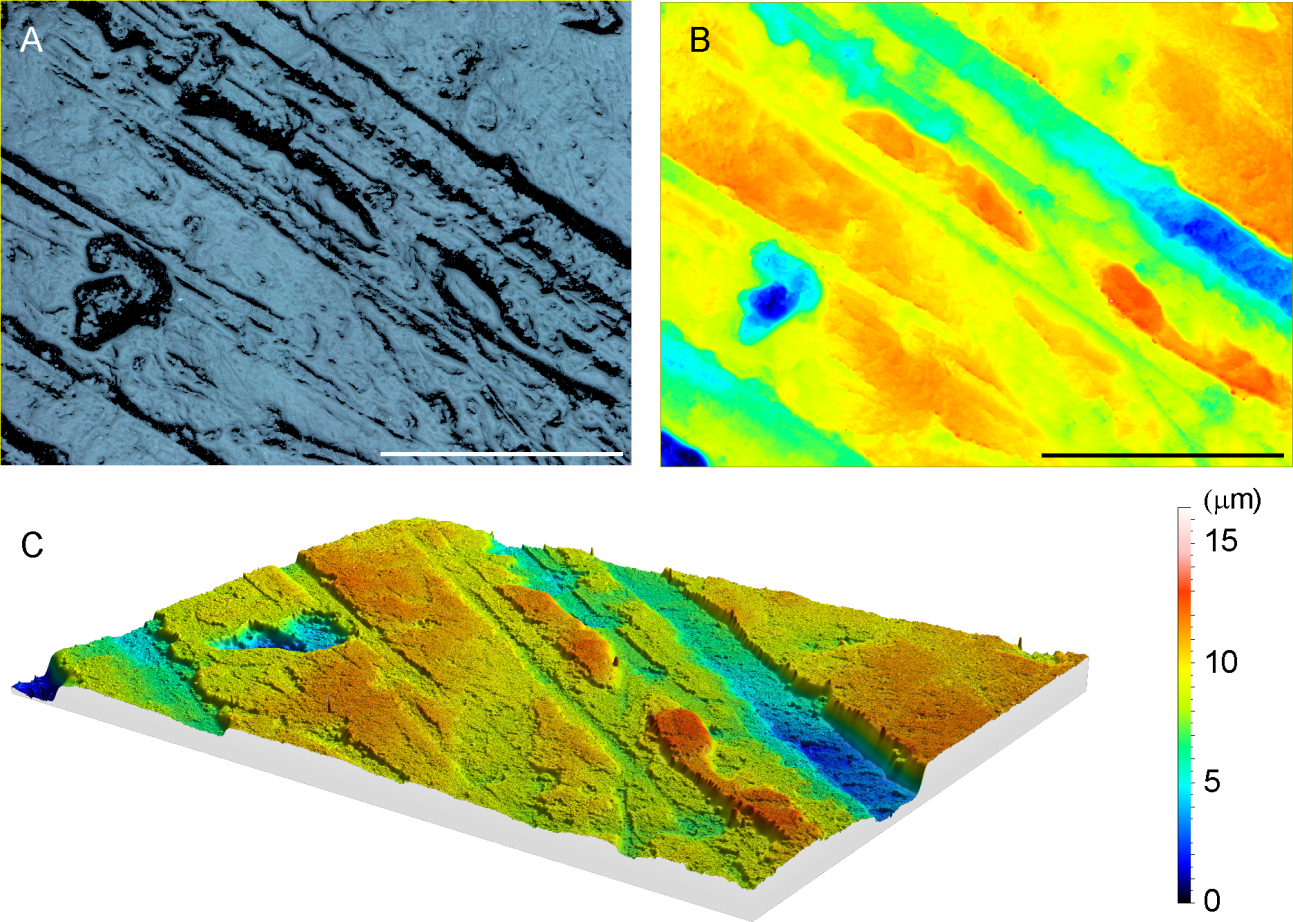
Close up of the dental microwear of *Exaeretodon* PVSJ 1091. Dental microwear within the area enclosed by the white rectangle in Fig 1C, scanned by the confocal laser microscope VK-9700 with a 100× objective lens to obtain ISO 25178 parameters. Scale bars are 100 μm. (A) Tooth surface texture image reconstructed from 3D coordinates. (B) Topography of tooth surface texture image. (C) Three dimensional view of tooth surface texture reconstructed from 3D coordinates. The color bar on the right side of Fig 3C shows the microwear surface topography in color.

Further, to measure the direction of scratches more objectively, in other words without the subjectivity of observer that accompanies a 2D microwear analysis [24,25], we calculated surface texture anisotropy from 3D data. For this analysis, an area of 261×192 μm (Figs 1 and 2) was scanned for each specimen using a 100× objective lens (numerical aperture = 0.73) with a voxel size of 0.137 μm for x and y axes, and 0.001 μm for z axis (Fig 3). The three dimensional coordinates obtained were uploaded to the surface roughness software (MountainsMap 7, ver. 7.4.8076, Digital Surf Co, France). Coordinates were mirrored in x and z axes to represent information of the real tooth surface. Surface data were leveled to remove the inclination of the specimen and the large-scale curvature of surface was removed using the form removal function of the MountainsMap 7 software (polynomial of increasing power = 2) and slopes for which angle steeper than 80 degrees were removed as outliers [26]. After these preparations, ISO 25178 parameters of texture anisotropy, Str and Std, were calculated using the MountainsMap 7. The Str value (no unit) takes value between 0 and 1. An isotropic surface shows the Str value close to 1, whereas strongly an anisotropic surface has values closer to 0. When texture is anisotropic, Std (degrees) suggests the direction of texture.

The angle of the scratches and the ISO parameter, Std, were corrected so that the direction of anterior becomes 90 degrees. Consequently, medial direction becomes 0 degree for the second postcanine of PVSJ 1091 and 180 degrees for postcanines of PVSJ 702 and 707. As postcanine teeth of *Exaeretodon* were positioned obliquely (Figs 1 and 2), in this study we used terms labial/lingual and mesial/distal when mentioning direction within a tooth, whereas lateral/medial and anterior/posterior are used to represent anatomical orientation of the cranium.

## Results

**Fig 4:**
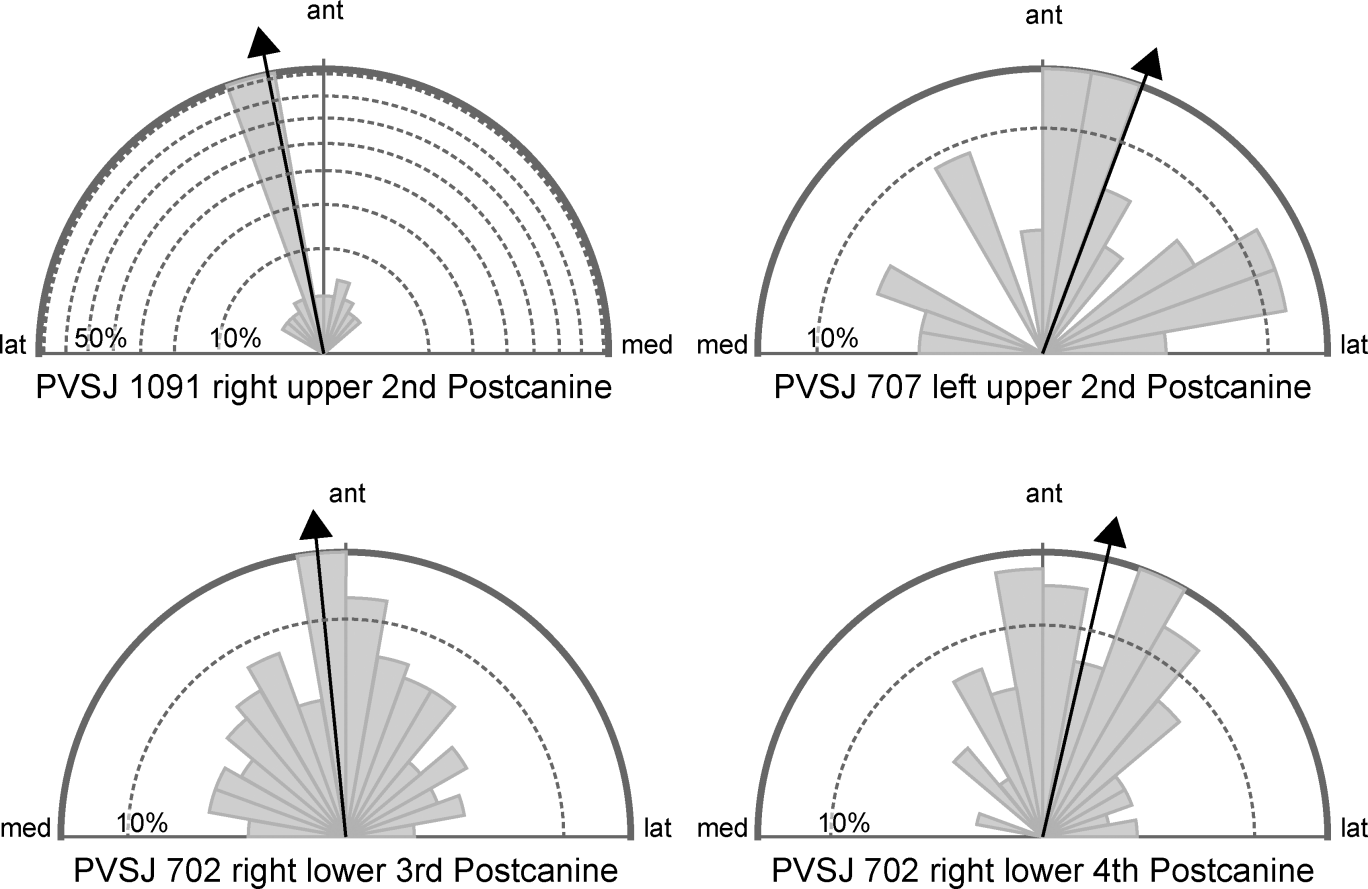
Rose diagrams and mean orientations of the scratches detected in the dental microware analysis of the four postcanines. Percentages of scratches in each 10 degrees orientation bin are shown by rose diagrams, with mean orientation shown as black arrows. In all four postcanines, mean orientations were within 20 degrees from the anterior direction. From the rose diagrams, it is clear that scratches on the second postcanine of PVSJ 1091 were well aligned, whereas scratches on the second postcanine of PVSJ 707 were more randomly distributed.

Mean orientations (mean vector) of the scratches were within 15 degrees from the anterior direction (90 degrees) for postcanines of PVSJ 1091 and 702, and within 20 degrees for the postcanine of PVSJ 707. Angular dispersion of PVSJ 1091 was the largest among four specimens and that of PVSJ 707 was the lowest. The 3D surface texture parameter Str recorded the lowest value in PVSJ 1091 and the highest in PVSJ 707. These results revealed scratches that were well aligned in PVSJ 1091 and its tooth surface was anisotropic, whereas scratches were more randomly distributed in PVSJ 707 and its surface texture is more isotropic (Fig 4). The 3D surface texture parameters Std were within 15 degrees from the anterior direction for all four postcanines. Mean orientation and Std were almost the same, differing less than 2 degrees in both the second postcanine of PVSJ 1091 and the third postcanine of PVSJ 702. These two angles differed about 20 and 24 degrees in the fourth postcainine of PVSJ 702 and the second postcanine of PVSJ 707, respectively. Mean scratch length is the longest in the second postcanine of PVSJ 1091 and shortest in the third postcanine of PVSJ 702, that of the fourth postcanine of PVSJ702 and the second postcanine of PVSJ 707 were intermediate and close to each other (Table 1).

**Table 1:** Positions of the four postcanine teeth on which microwear were preserved, and results of the dental microwear analysis. Angular dispersion and Str has values between 1 and 0. Angular dispersion close to 1 indicates scratches are well aligned. Str value close to 0 indicate strongly anisotropic surface texture and Std gives the direction of texture when the texture is anisotropic. For angular measurements, anterior direction was set at 90 degrees.

Specimen number	PVSJ 1091	PVSJ 702	PVSJ 702	PVSJ 707
Positon of teeth row	Upper (R)	Lower (R)	Lower (R)	Upper (L)
Tooth position	2nd PC	3rd PC	4th PC	2nd PC
Occlusal surface	Yes	Yes	Yes	No
Number of scratches	59	201	87	32
Mean orientation (degree)	101.9	96.1	77.7	70.2
(95% confidence intervals)	(98.0–106.0)	(90.5–101.4)	(72.2–83.7)	(52.7–88.2)
Angular dispersion	0.96	0.79	0.88	0.71
Mean length (μm)	437.6	84.5	160.6	177.7
(95% confidence intervals)	(363.5–523.5)	(77.4–92.6)	(142.5–181.6)	(150.8–217.1)
Str	0.110	0.240	0.247	0.316
Std (degree)	103.0	95.7	98.1	93.9

Abbreviations: L, left; PC, postcanine; R, right

## Discussion and conclusions

Both mean orientation of scratches and direction of texture anisotropy (Std) revealed that the scratches of *Exaeretodon argentinus* were directed roughly in the antero-posterior direction. We found that scratches on the occlusal surfaces were more aligned and directed closer to the antero-posterior direction compared with that of the non-occlusal surface on the internal cusp of second postcanine of PVSJ 707. The results of 2D microwear analyses and the values of ISO 25178 parameters are consistent with each other. The highest angular dispersion and the lowest isotropy (Str) both indicate the scratches observed on the second postcanine of PVSJ 1091 are well-aligned. The lowest angular dispersion and the highest isotropy observed in the second postcanine of PVSJ 707 indicate its scratches are oriented more randomly. The mean direction of orientation of scratches and Std are close to each other, especially when Str is low, in other word, when the scratches are well-aligned.

Crompton [2] assumed that shearing occurred between the distal surface of the transverse ridge of upper postcanine and the mesial surface of the transverse ridge of lower postcanine during the first half of the palinal masticatory movement of the mandible, and between the mesial part of an adjacent distal upper postcanine and the transverse ridge of the lower postcanine during the second half of the palinal masticatory movement of the mandible (Fig 1B). Following this palinal movement, Goñi and Goin [6] assumed an additional medial movement of the mandible causing dynamic occlusion between the internal cusp of the upper postcanine and occlusal basin of lower postcanine. Orientation of scratches on the occlusal surfaces that were in the antero-posterior direction (Fig 4) support the existence of palinal jaw movements proposed by Crompton [2]. On the other hand, scratches on the internal cusp of the second upper postcanine of PVSJ 707 that were mainly directed in anteroposterior direction and absence of scratches that were directed mediolaterally on the transverse ridge of second upper postcanine of PVSJ 1091 do not support the existence of a medial movement of the mandible that followed the palinal movement in the masticatory jaw movement of *Exaeretodon argentines* (Fig 4). The medial movement of the mandible must have produced mediolaterally directed scratches on these tooth surfaces as the internal cusp of the upper postcanine was passing against the lingual edge of the antagonistic lower postcanine and the transverse ridge was passing against the labial rim of the antagonistic lower postcanine ([6] Fig 3). Further, the medially-directed mandibular movement assumed by Goñi and Goin [6] is unlikely without the mobility in the mandibular symphysis, because without adequate mobility, medial movement on one side of the jaw would cause lateral movement of the other, which is prevented by the ventrally projecting labial lobe of the upper postcanine in *Exaeretodon* (Fig 1B).

The mean orientation of scratches was deviates by 10–20 degrees laterally from antero-posterior direction (Fig 4) except for the third postcanine of PVSJ 702, which deviates six degrees medially. These slight deviations reflect either the real jaw movement or postmortem distortion of the specimen and/or measurement error. Mean orientations of the scratches in the adjacent postcanines, third and fourth postcanines of mandible of PVSJ 707, differed about 18 degrees, which is unlikely to reflect the real jaw movement. Thus these deviations are more likely to occur from postmortem distortions and/or measurement error. As the orientation of scratches were measured based on the assumption that tooth surfaces are horizontal, inclinations of tooth surfaces from the horizontal plane can cause measurement errors. To reduce measurement errors, the microwear results need to be reconstructed in 3D, in which the inclination of the tooth surfaces is measured from the original specimen and is taken into account when the three dimensional direction of the scratches are calculated [28].

Another feature of dental microwear worth mentioning are the wide scratches seen in PVSJ 1091 (Fig 2). Of the 59 scratches in PVSJ 1091, 32 could be measured, which averaged 9.8 μm in width. In contrast, scratches in other specimens were not as wide as that of PVSJ 1091, probably were less than 2 μm wide and difficult to measure given the resolution of images at hand. Scratch width of modern artiodactyls [29] or primates [30] are reported to be around 1–2 μm. Thus, compared with modern herbivorous mammals of similar body sizes, scratches of PVSJ 1091 seemed much wider, whereas that of other specimens were comparable. Furthermore, the lengths of the scratch in PVSJ 1091 were much longer than in the other specimens (Table 1). These variations among specimens may be due to positional differences of the tooth surfaces where microwear was analyzed. Given the transverse ridge of the postcanine of PVSJ 1091 where the mold was taken, happened to be the main shearing surface of *Exaeretodon* [2], scratches in PVSJ 1091 may be wider and longer than scratches of other specimens, because they were either taken from lingual rims of lower postcanines or from the non-occlusal internal cusp of upper postcanine. It is also possible that differences in diet and environment between the different *Exaeretodon* specimens may have contributed to the observed differences in width and length of the scratches. Caution is needed when drawing inference on the reasons for such extremely wide scratches of PVSJ 1091, because both masticatory jaw movements and enamel structures differ between modern mammals and *Exaeretodon*, which enamel type is non-prismatic [31]. These factors, in combination with the size of the abrasive particles ingested during mastication, affects scratch width in a complicated manner. For example, larger abrasive particle left wider scratch on non-prismatic enamel surface, but not on prismatic enamel surface [32]. However, it is likely that the contamination of large abrasives, such as grits, occurred when PVSJ 1091 foraged. From the current evidence, it is not clear that contamination of abrasives during mastication is unique to PVSJ 1091, or it is common among *Exaeretodon*.

Discussion of the result of this study in light of the phylogeny of eucynodonts [4,33] and the two other microwear studies of cynognathian cynodonts [15,17] supports the hypothesis that palinal masticatory jaw movement evolved within the cynognathian cynodonts, from the wholly orthal jaw movement of basal *Diademodon* to the palinal movement seen in derived *Exaeretodon* and *Dadadon*. Dental microwear described here indicates lateral jaw movement was unlikely to have been evolved among the cynognathian cynodonts. These findings highlight the utility of dental microwear in revealing the evolution of jaw movement among cynodonts.

## Supporting information

S1 Table Angle, length and width of scratches. (XLSX)
